# Recurrent toxic epidermal necrolysis with two different drugs in a patient with chronic kidney disease–A case report with an atypical presentation

**DOI:** 10.1002/ccr3.8027

**Published:** 2023-10-09

**Authors:** Mahesh Mathur, Neha Thakur, Sunil Jaiswal, Srijana Maharjan, Supriya Paudel, Anjali Shrestha

**Affiliations:** ^1^ Department of Dermatology, Venereology, and Leprology College of Medical Sciences and Teaching Hospital Bharatpur Nepal

**Keywords:** chronic kidney disease, cross‐reactivity, recurrent toxic epidermal necrolysis

## Abstract

Toxic epidermal necrolysis (TEN) is a dermatologic emergency usually attributed to drugs. Recurrent episodes of TEN are more common in the pediatric population than in adults. Patients carrying susceptible specific haplotypes, cross‐reactivity between the drugs, and drug metabolites generated by the Cytochrome P450 are the key factors for the recurrent episodes. Chronic kidney disease (CKD) increases the risk of toxic epidermal necrolysis by altering the pharmacokinetics and pharmacodynamics of the drug with comparatively higher mortality in this group of patients. We hereby present an elderly female with 2 episodes of TEN following intake of furosemide at present and Nimesulide 3 years back. Cross‐reactivity between these drugs because of the similar stereochemical structure might have triggered the second episode. The second episode of TEN was milder in presentation with a short latency period without any constitutional symptoms as compared to the first episode. Thus, treating physicians should always consider cross‐reactivity between the chosen drugs in order to prevent subsequent life‐threatening episodes, especially in patients with CKD.

## Graphical abstract

Recurrent episodes of toxic epidermal necrolysis (TEN) in a 60‐year‐old female patient with chronic kidney disease. A–C: Episode of TEN 3 years back following intake of Nimesulide. D–F: Episode of TEN at present following intake of Furosemide.

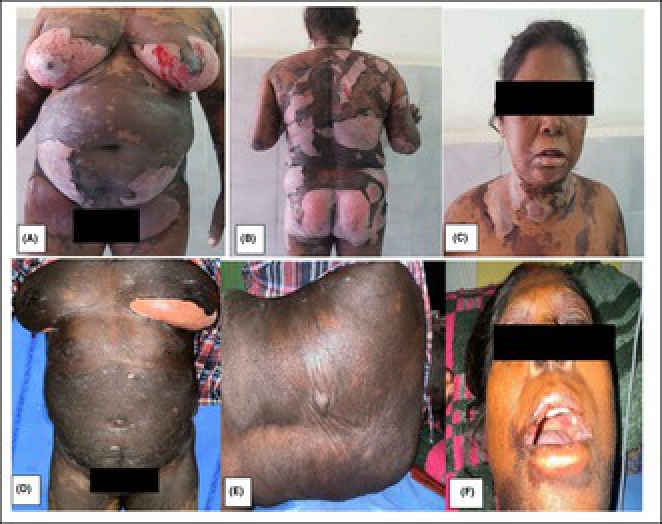

## 1 INTRODUCTION

Toxic epidermal necrolysis (TEN) is a life‐threatening mucocutaneous disorder characterized by diffuse erythema, necrosis, and flaccid bulla involving body surface area (BSA) ≥ 30% and resulting in desquamation. The incidence of TEN is 1–2/1000000 annually.^1^ The majority of the cases of TEN are drug‐induced.^2^

Chronic kidney disease is defined as glomerular filtration rate <60 mL/min/1.73 m^2^ for 3 months or more, regardless of cause.^3^ Around 25.7%–38% of patients with chronic kidney disease develop SJS/TEN as reported in the literature.^4^, ^5^

Recurrent episodes of TEN are rare in adults, however, reported more frequently in the pediatric population.^2^ We hereby report a case of recurrent TEN in a patient with chronic kidney disease with two different drugs.

## 2 CASE PRESENTATION

**FIGURE 1:**
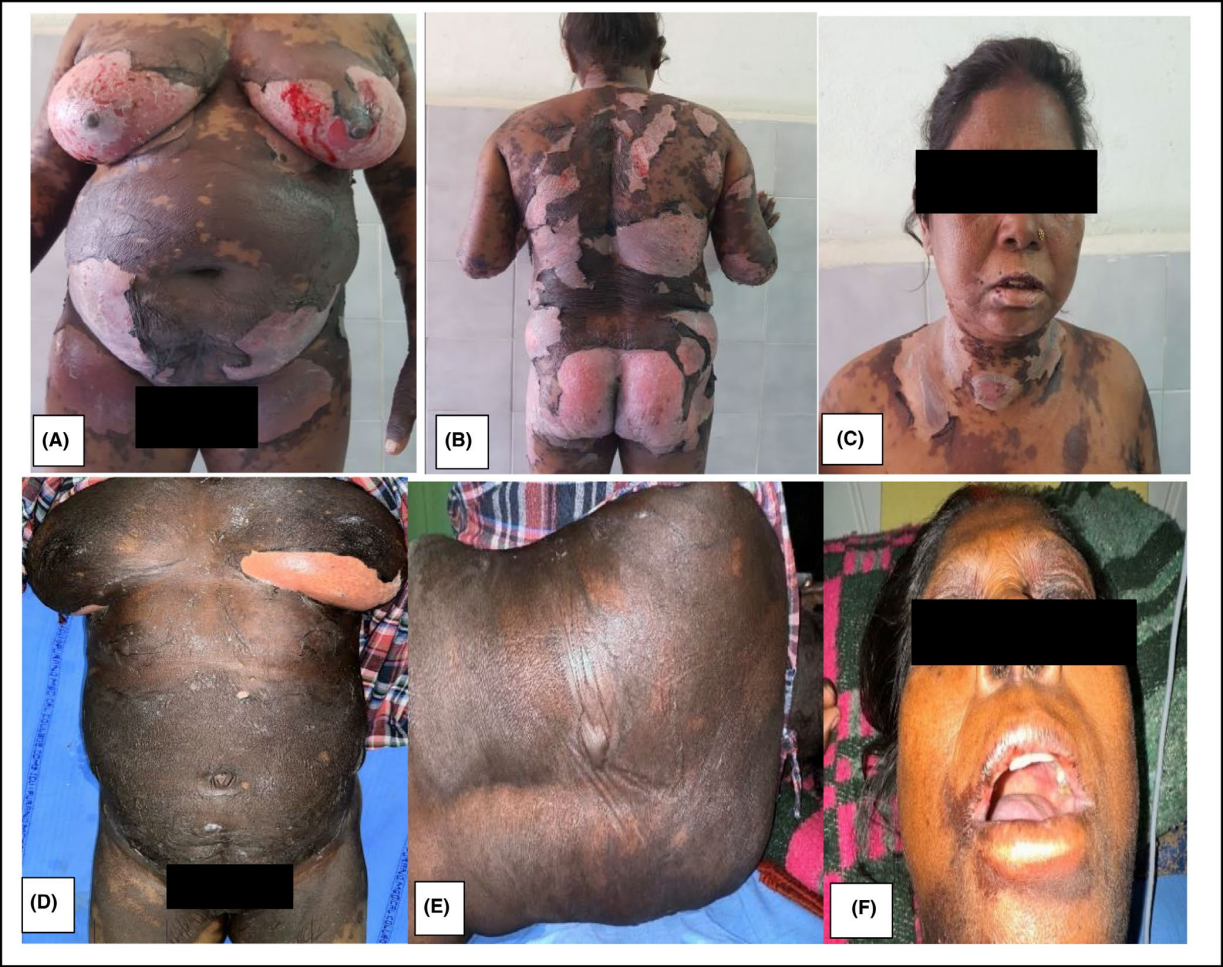
A‐C shows mucocutaneous involvement in the first episode of TEN 3 years back which was more severe as compared to the second (recent) episode as shown in D‐F.

A 60‐year‐old female, a known case of chronic kidney disease secondary to hypertension, presented to the Emergency Department with generalized ill‐defined confluent dusky erythematous tender patches and flaccid bullae on the trunk and extremities with peeling of skin over the mammary region for 3 days (Figure 1D–F). She had erosions on her lips, hard palate, and mucopurulent discharge from the eyes. Skin lesions developed after 16 h of intake of the last dose of furosemide as advised by the treating physician for facial puffiness and pedal edema.

Nikolsky's sign was positive. The BSA involved was approximately 40% and she was hemodynamically stable. Baseline investigations revealed raised serum BUN (106.6 mg/dL), raised creatinine (4.6 mg/dL), and hypokalemia (2.9 meq/L). Arterial blood gas analysis showed decreased pH (7.24) and decreased bicarbonate levels (12 meq/L).

**FIGURE 2:**
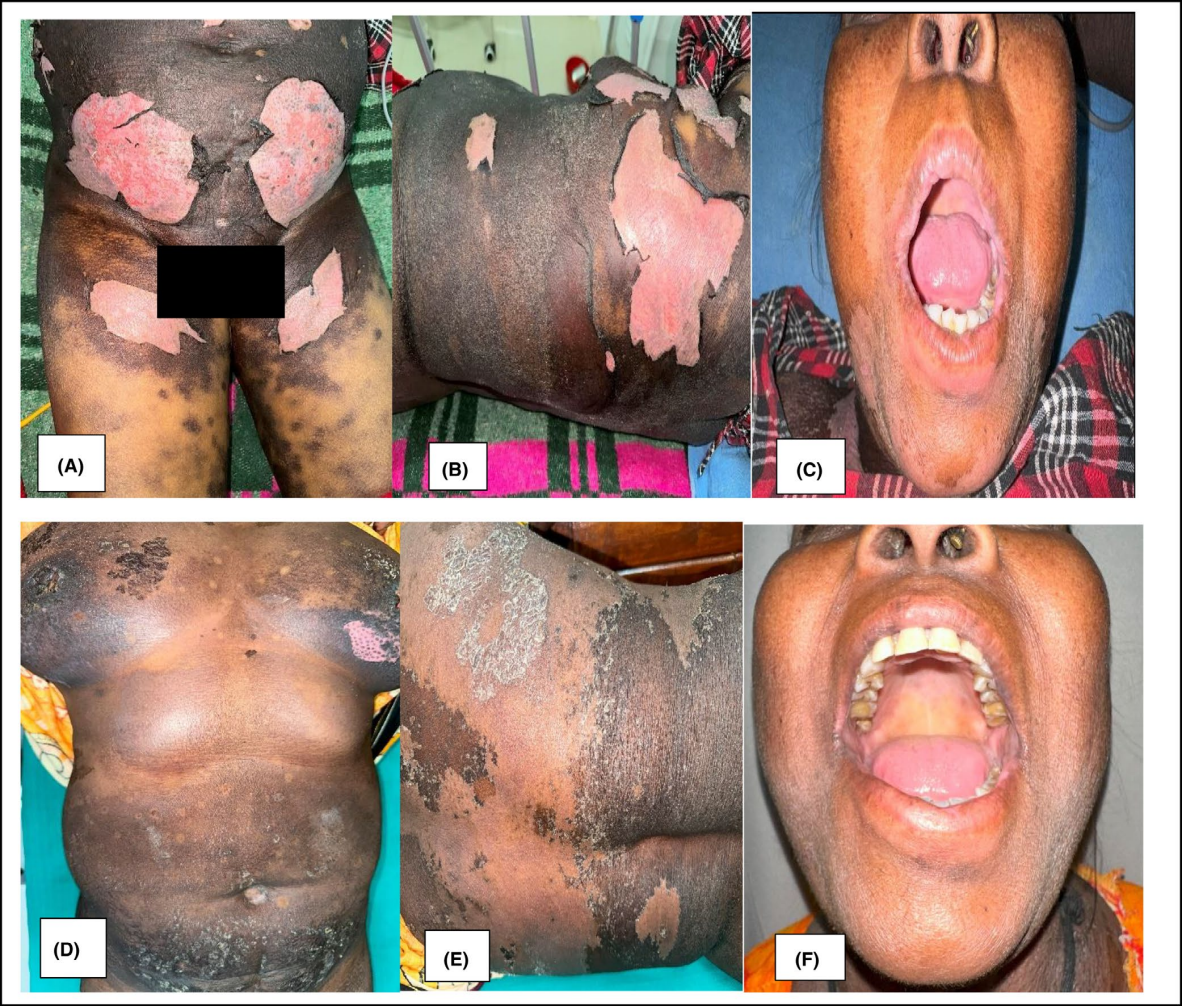
A‐C shows initial reepithelialization over the denuded areas on the 7th day, complete on the 15th day of admission as shown in D‐F along with the complete resolution of the oral lesion.

Diagnosis of TEN with acute on chronic kidney disease and Metabolic Acidosis was made with a baseline SCORTEN score of 5. Furosemide was stopped. Patient was admitted to the ICU and was administered intravenous fluids, antibiotics, and a short course of parenteral hydrocortisone along with mucocutaneous care. Vitals, urine output, serum electrolytes, and creatinine were monitored. SCORTEN score was 5 on the third day of admission. Initial reepithelialization of skin lesion was observed after 7 days (Figure 2A–C) and was completed by Day 15 (Figure 2D–F). Serum creatinine levels decreased to 3.2 mg/dL, however, it did not recover completely.

The patient had a similar episode of skin lesions 3 years back following intake of Nimesulide for myalgia and developed extensive mucocutaneous lesions as depicted in Figure 1A–C. She was admitted to our hospital during the previous episode and was managed successfully.

## 3 DISCUSSION

TEN is a dermatological emergency mediated predominantly by CD8^+^ T lymphocytes and is associated with an average mortality of 25%.^2^, ^6^ The recent understanding of epidermal necrolysis in TEN is thought to be an immune‐driven pathway mediated by granulysin released by drug‐specific cytotoxic CD8 T cells and natural killer cells.^6^, ^7^

Chronic kidney disease increases the risk of adverse drug reactions by changing drug metabolism via altering renal and nonrenal clearance, protein binding, and volume of distribution. Chronic kidney disease has also been found to be a significant strong, independent, prognostic factor for the mortality of patients with SJS/TEN apart from the parameters of SCORTEN.^5^ The mortality rate in patients with chronic kidney disease who develop SJS/TEN is comparatively higher and is estimated to be approx. 43.7% which was observed in a study conducted by Hung et al.^4^

Patients carrying susceptible specific haplotypes, cross‐reactivity between the drugs with very similar stereochemical structures, and drug metabolites generated by the Cytochrome P450 enzyme complex may influence the recurrence of SJS /TEN.^2^ Our patient had two episodes of TEN following intake of Furosemide at present and Nimesulide 3 years back.

The intense literature search revealed a similar chemical structure in Nimesulide and Furosemide. Nimesulide is a cyclooxygenase‐2 inhibitor having phenyl and 2‐methylsulfonamido‐5‐nitrophenyl groups.^8^ Similarly, Furosemide is a diuretic which is a chlorobenzoic acid that consists (furan‐2‐ylmethyl) amino and a sulfamoyl group.^9^ Both drug contains the sulfonamide functional group (R−S(=O)_2_−NR_2_) which might have triggered the second episode in our patient.

In our patient, the skin lesions in the second episode of TEN appeared earlier as compared to the usual duration reported in the literature. The cause of the short latency period may be attributed to the sensitization of the patient to the sulfonamide group of drugs in the first episode. Similarly, our patient lacked constitutional symptoms and the mucocutaneous involvement was less severe as compared to the first episode as depicted in Figure 1, which might be due to the early presentation of the patient to our center along with early therapeutic intervention. Patient was finally advised to avoid sulpha group containing drugs to prevent the subsequent episodes in the future.

## 4 CONCLUSION

Conventionally, nimesulide and furosemide are two different groups of drugs but an intense literature search revealed that both drugs have a common sulfonamide functional group which triggered the subsequent episode of TEN in our patient, in the setting of chronic kidney disease. Thus, it is important to note that any patient who has had a previous hypersensitivity reaction to a drug is at high risk for recurrence with another drug having a similar chemical structure, and treating physicians should always consider cross‐reactivity between the chosen drugs in order to prevent the subsequent life‐threatening episodes, especially in patients with chronic kidney disease as the mortality rate is comparatively higher in these group of patients.

## AUTHOR CONTRIBUTIONS

**Mahesh Mathur:** Conceptualization; data curation; formal analysis; supervision; writing – original draft; writing – review and editing. **Neha Thakur:** Conceptualization; investigation; methodology; supervision; validation. **Sunil Jaiswal:** Conceptualization; data curation; formal analysis; validation; writing – original draft; writing – review and editing. **Srijana Maharjan:** Investigation; methodology; validation; visualization. **Supriya Paudel:** Conceptualization; formal analysis; methodology; supervision. **Anjali Shrestha:** Data curation; formal analysis; investigation; visualization.

## CONFLICT OF INTEREST STATEMENT

The authors declare no conflicts of interest.

## ETHICS STATEMENT

The patient in this manuscript has given written informed consent for the use of their case details (including photographs) for publication.

## 5 CONSENT

Written informed Consent has been taken from the patient.

## Data Availability

The data that support the findings of this study are openly available in (repository name e.g., “figshare”) at http://doi.org/10.1002/ccr3.8027, reference number (Article ID: CCR38027).

## References

[1] Harr T , French LE . Toxic epidermal necrolysis and Stevens–Johnson syndrome. Orphanet J Rare Dis. 2010;5(39):1‐11. 21162721 10.1186/1750-1172-5-39 PMC3018455

[2] Drateln CR , Gomez‐Hernandez N , Martinez NR , Torres‐Lozano C . Recurrent toxic epidermal necrolysis syndrome: a report of two cases. Drug Saf Case Rep. 2016;3(9):1‐4. 27747689 10.1007/s40800-016-0032-x PMC5021638

[3] Levey AS , Eckardt KU , Tsukamoto Y , et al. Definition and classification of chronic kidney disease: a position statement from kidney disease: improving global outcomes (KDIGO). Kidney Int. 2005;67(6):2089‐2100. 15882252 10.1111/j.1523-1755.2005.00365.x

[4] Wasuwanich P , So JM , Chakrala TS , Chen J , Motaparthi K . Epidemiology of Stevens–Johnson syndrome and toxic epidermal necrolysis in the United States and factors predictive of outcome. JAAD Int. 2023;13:17‐25. 37575514 10.1016/j.jdin.2023.06.014 PMC10413346

[5] Hung CC , Liu WC , Kuo MC , Lee CH , Hwang SJ , Chen HC . Acute renal failure and its risk factors in Stevens–Johnson syndrome and toxic epidermal necrolysis. Am J Nephrol. 2009;29(6):633‐638. 19155617 10.1159/000195632

[6] Kinoshita Y , Saeki H . A review of the pathogenesis of toxic epidermal necrolysis. J Nippon Med Sch. 2016;83(6):216‐222. 28133001 10.1272/jnms.83.216

[7] Gupta LK , Martin AM , Agarwal N , et al. Guidelines for the management of Stevens–Johnson syndrome/toxic epidermal necrolysis: an Indian perspective. Indian J Dermatol Venereol Leprol. 2016;82:603‐625. 27716721 10.4103/0378-6323.191134

[8] National Center for Biotechnology Information. PubChem Compound Summary for CID 4495, Nimesulide[Internet].US:NLM. 2023 Available from:https://pubchem.ncbi.nih.gov/compound/Nimesulide

[9] National Center for Biotechnology Information. PubChem Compound Summary for CID 3440, Furosemide[Internet].US:NLM. 2023 Available from:https://pubchem.ncbi.nih.gov/compound/Furosemide

